# Traditional Chinese medicine in collaboration with conservative conventional medicine for lumbar herniated intervertebral disc: A protocol for systematic review and meta-analysis

**DOI:** 10.1097/MD.0000000000025652

**Published:** 2021-04-23

**Authors:** Hyungsuk Kim, Won-Seok Chung

**Affiliations:** Department of Clinical Korean Medicine, Graduate School, Kyung Hee University, Kyungheedae-ro, Dongdaemun-gu, Seoul, Korea.

**Keywords:** conservative conventional medicine, lumbar herniated intervertebral disc, protocol, systematic review, traditional Chinese medicine

## Abstract

**Background:**

Lumbar herniated intervertebral disc (LHIVD) is a common disease that causes low back pain. Traditional Chinese medicine (TCM) with conservative conventional treatment is known to be effective at treating LHIVD, but evidence for complex TCM therapies with conventional intervention has not been sufficiently collected to facilitate quality assessment and synthesis of data.

**Methods and analysis:**

Studies were retrieved from the following databases: the Cochrane Central Register of Controlled Trials, EMBASE, MEDLINE/PubMed, 7 Korean databases (Korean Studies Information Service System, Korean Traditional Knowledge Portal, Oriental Medicine Advanced Searching Integrated System, Korean National Assembly Digital Library, Korean Association of Medical Journal Editors, National Digital Science Library, and Database Periodical Information Academic), Japan Medical Abstracts Society, and Chinese National Knowledge Infrastructure. The risk of bias of the included studies will be assessed using the Cochrane Assessment Tool for Risk of Bias. Eligible studies were quantitatively synthesized through a meta-analysis. The primary outcome will be pain scales, and the secondary outcomes will include range of motion, questionnaires for lumbar function, questionnaire for quality of life, etc.

**Ethics and dissemination:**

Ethical approval was waived for this study protocol because it does not provide any patient data. The results of this review will be disseminated through peer-reviewed publications.

**Registration number:**

DOI 10.17605/OSF.IO/K7NJ8 (https://osf.io/k7nj8).

## 1 Introduction

Lumbar herniated intervertebral disc (LHIVD) is one of the main causes of lower back and leg pain, accounting for about 5% of lower back pain patients.^[1]^ With changes in lifestyle patterns to frequent sitting, an increasing number of people are susceptible to lower back pain and LHIVD problems. If not properly treated, the symptoms of LHIVD can progress to a more serious stage, even resulting in muscular atrophy, motor disturbance, and urinary and fecal incontinence. This affects patients’ quality of life and their ability to work. Therefore, it is necessary to treat LHIVD with effective conservative methods in the early stages, thus protecting patients from progressing to conditions that require surgery.^[2]^

Traditional Chinese medicine (TCM), which includes widely used treatments with a long history of use, has proven to be effective for treating various kinds of diseases, especially musculoskeletal disorders.^[3]^ For LHIVD, there have been many systematic reviews^[4–6]^ and clinical practice guidelines for LHIVD,^[7]^ but these studies only focused on one method of TCM treatment. In this systematic review, we aimed to assess and evaluate the efficacy of 2 or more TCM treatments in combination with conservative conventional treatment.

## 2 Methods

### 2.1 Study registration

This protocol is described according to the preferred reporting items for systematic reviews and meta-analysis (PRISMA) protocols,^[8]^ which have been registered on the Open Science Framework (osf.io/k7nj8).

### 2.2 Eligibility criteria for study selection

#### 2.2.1 Types of studies

Only randomized controlled trials (RCTs) will be included in this systematic review, whereas quasi-RCTs and crossover studies will not be included. There will be no restrictions on the language of the study. Both printed and ongoing studies will be included.

#### 2.2.2 Types of participants

Patients diagnosed as LHIVD without operative process will be included. There will be no restriction in age and sex.

#### 2.2.3 Types of interventions

The experimental group must include 2 or more TCM treatments, including acupuncture. As long as the needle penetrates the skin, it will be considered as acupuncture, excluding acupressure or laser acupuncture. Tuina in this review will not include any forms of manipulation, except for lumbar traction. If the group has been treated with TCM plus conservative conventional intervention, the same conventional intervention should be applied to the control group. For the comparison group, all kinds of oral medications, injections, physiotherapy, etc will be included.

#### 2.2.4 Types of outcome measures

The primary outcome will be pain scales, such as the numerical rating scale and visual analogue scale. The secondary outcome will be the range of motion of the lumbar spine, questionnaires for lumbar function, questionnaires for quality of life, etc.

### 2.3 Search strategy

#### 2.3.1 Electronic data

The Cochrane Central Register of Controlled Trials, EMBASE, MEDLINE/PubMed, 7 Korean databases (Korean Studies Information Service System, Korean Traditional Knowledge Portal, Oriental Medicine Advanced Searching Integrated System, Korean National Assembly Digital Library, Korean Association of Medical Journal Editors, National Digital Science Library, and Database Periodical Information Academic), the Japan Medical Abstracts Society, and the Chinese National Knowledge Infrastructure will be systematically searched from their inception to February 2021. The search methods are presented in Table 1.

**Table 1:** Search strategies for PubMed.

#1	herniated disc OR herniated disk OR disc prolapse OR disk prolapses OR disc prolapses OR disk prolapse OR intervertebral disc displacement OR intervertebral disc displacements OR intervertebral disk displacement OR intervertebral disk displacements OR slipped disc OR prolapsed disc OR prolapsed disk
#2	acupuncture OR acupuncture OR acupuncture therapy OR Chinese medicine OR Chinese herbal medicine OR Chinese medicine OR medicine, Chinese traditional OR traditional Chinese medicine OR herbal medicine OR botanical medicine OR ethnobotanical medicinal use OR ethnobotanical medicine OR ethnobotanical remedy OR herb medicine OR herbal medicine OR herbal remedy OR medicine, herbal OR phyto-medicine OR phytomedical remedy OR phytomedicine OR plant medicine OR plant-based medicine OR plant-based remedy OR tui na OR tuina OR pharmacopuncture OR herb acupuncture OR herbal acupuncture OR herbalized acupuncture OR pharmaco-acupuncture OR pharmaco-puncture OR pharmacoacupuncture OR pharmacopuncture OR electroacupuncture OR acupuncture, electric OR electric acupuncture OR electrical acupoint stimulation OR electrical acupuncture OR electro-acupuncture OR electroacupuncture OR electrode acupuncture OR electronic acupuncture OR moxibustion OR moxibustion OR warm acupuncture OR cupping therapy OR cupping (therapy) OR cupping manipulation OR cupping therapy OR cupping treatment OR fire cupping OR flash cupping OR moving cupping OR suction cupping OR vacuum cupping
#3	randomized controlled trials OR randomized controlled trial OR controlled trial, randomized OR randomized controlled study OR randomized controlled trial OR randomized controlled study OR randomized controlled trial OR trial, randomized controlled
#4	#1 AND #2 AND #3

#### 2.3.2 Search for other resources

To search the literature more elaborately, we will look up the references of the selected papers. For literature that is not uploaded online, manual searching will be conducted.

### 2.4 Data collection and analysis

#### 2.4.1 Study selection

**Figure 1:**
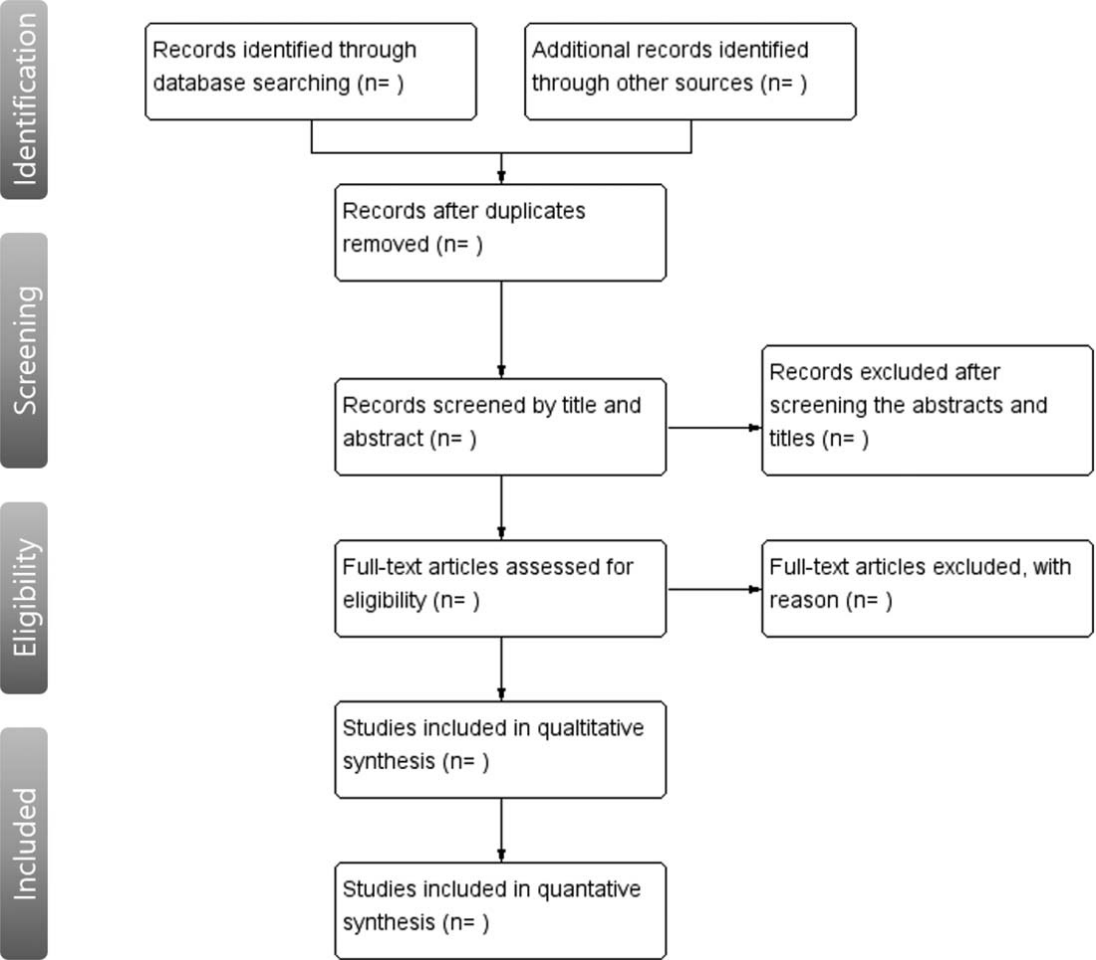
flowchart of the systematic review.

The search strategy will be independently applied by 2 reviewers. Inclusion and exclusion criteria were judged for selection. When there is no agreement between the reviewers, other authors will discuss the consensus. A flowchart for the selection process based on the PRISMA flow chart is presented in Figure 1.

#### 2.4.2 Data extraction and management

A specially designed form was used to extract data from selected articles by 2 reviewers. The contents of the form will be; basic information of the participants, the onset and diagnosis date of LHIVD, type and frequency of intervention in the experimental and control groups, types and time points of outcome assessments, and adverse events. In an ambiguous situation, another reviewer will discuss the decision.

#### 2.4.3 Assessment of the risk of bias and quality of the included studies

Two independent reviewers will assess the risk of bias using the tool developed by the Cochrane Collaboration group.^[9]^ Seven domains will be evaluated: random sequence generation, allocation concealment, blinding of participants and personnel, blinding of outcome assessment, incomplete outcome data, selective outcome reporting, and other sources of bias. Each domain will be assessed as either “high risk,” “low risk,” or “unclear risk.” If 2 reviewers have inconsistent opinions in the same domain of an article, a third reviewer will mediate the discordance.

#### 2.4.4 Assessment of the effect of treatment

Continuous data will be analyzed using mean differences and 95% confidence intervals. Weighted mean differences will be adopted in the presence of the same scales. If not, standardized mean differences will be used.

#### 2.4.5 Management of missing data

If there are missing data or ambiguous information in the literature, we will contact the corresponding author for explanation and clarity. However, when needed information is not obtained, our efforts will not be used in our study.

#### 2.4.6 Data synthesis

When data can be synthesized, a meta-analysis will be performed using the software distributed by the Cochrane Collaboration (Review Manager Software Version 5.3). When heterogeneity is sufficiently high (*I*^*2*^ > 50%), a random-effects model will be employed, whereas a fixed-effects model will be used otherwise. Subgroup analysis will be considered with *I*^*2*^ greater than 75%, because it indicates very high heterogeneity.

#### 2.4.7 Subgroup analysis

The criteria for subgroup analysis are as follows:

1. The type of TCM interventions (e.g., Acupuncture + herbal medicine, acupuncture + moxibustion, acupuncture + Tuina, acupuncture + more than one TCM method).
2. Duration of TCM intervention
3. Period of follow-up.
4. Type of acupuncture (manual acupuncture, electroacupuncture, pharmacoacupuncture, etc).

#### 2.4.8 Ethics and dissemination

This is a protocol for systematic review and there is no patient information in the current manuscript; therefore, ethical approval is not needed. The findings of this systematic review will be disseminated through journals or presentations at conferences.

## 3 Discussion

LHIVD is a common disease that causes lower back pain and radiating leg pain. Successful conservative treatment is key to keeping LHIVD patients from progressing to more severe conditions and to return to their normal daily life. RCTs and systematic reviews have been published regarding the effectiveness of TCM and conventional care; however, no systematic reviews have yet determined the efficacy of complex TCM therapies in collaboration with conventional therapy. This systematic review will provide clinicians with insights into the effective and efficient treatment of LHIVD patients.

## Author contributions

**Conceptualization:** Won-Seok Chung.

**Data curation:** Hyungsuk Kim.

**Formal analysis:** Hyungsuk Kim, Won-Seok Chung.

**Funding acquisition:** Won-Seok Chung.

**Project administration:** Hyungsuk Kim, Won-Seok Chung.

**Writing – original draft:** Hyungsuk Kim.

**Writing – review & editing:** Hyungsuk Kim, Won-Seok Chung.
